# Comparative Phytochemical Characterization of Turkish Propolis Extracts and Their Antioxidant, Antibacterial, and Anticancer Activities

**DOI:** 10.3390/molecules31173071

**Published:** 2026-08-31

**Authors:** Serhat Karabıcak, Oktay Bıyıklıoğlu, Şeymanur Aktaş, Derya Keleşoğlu Kalemkaş, Demet Erdağ, Tarık Mecit, Selin Çeter, Idris Yazgan, Talip Çeter

**Affiliations:** 1 Department of Biology, Faculty of Science, Kastamonu University, Kastamonu 37150, Türkiye; serhatkarabicak@gmail.com (S.K.); seyymanuraktas@gmail.com (Ş.A.); derykls37@gmail.com (D.K.K.); akdoganselin@gmail.com (S.Ç.); iyazgan@kastamonu.edu.tr (I.Y.); talipceter@gmail.com (T.Ç.); 2 Department of Biophysics, Faculty of Medicine, Bilecik Seyh Edebali University, Bilecik 11100, Türkiye; erdagdem@gmail.com; 3 Department of Physiology, Faculty of Medicine, Biruni University, İstanbul 34025, Türkiye; tmecit@biruni.edu.tr

**Keywords:** propolis, palynological analysis, chemical composition, antibacterial, anticancer

## Abstract

Geographical and botanical origin dominate the chemical composition and biological activity of propolis samples. In this study, thirty Turkish propolis samples collected from different regions within the Black Sea region were comprehensively evaluated to investigate the relationships between phytochemical composition, antioxidant capacity, antibacterial activity, and anticancer potential. The propolis samples were extracted using 70% aqueous ethanol and labeled as K1-K30. Total phenolic content (TPC), total flavonoid content (TFC), ferric reducing antioxidant power (FRAP), and DPPH radical scavenging activity were determined. Antibacterial activity was evaluated against both Gram-positive and Gram-negative bacterial strains using inhibition zone (ZI), minimum inhibitory concentration (MIC), and minimum bactericidal concentration (MBC) tests. Most of the extracts showed measurable antibacterial activity, where Gram-positive bacteria generally showed higher susceptibility compared to the tested Gram-negative bacterial species. The extracts K12, K13, K22, K24, and K28 demonstrated the strongest broad-spectrum antibacterial activity. Pearson correlation analysis revealed that antibacterial efficacy was more strongly associated with antioxidant capacity and flavonoid richness than with total phenolic content alone. FRAP and TFC emerged as the strongest predictors of antibacterial activity, while specific phenolic compounds (particularly quercetin and trans ferulic acid) were associated with enhanced activity against Gram-negative bacteria. Preliminary anticancer activity was assessed using cell viability assays at 24, 48, and 72 h. Cytotoxic responses were highly heterogeneous and strongly time-dependent. K10, among the extracts, exhibited the strongest anticancer activity, showing inhibition rates above 80% at 24 and 48 h, whereas K4 and K23 also demonstrated considerable cytotoxic effects. Notably, antibacterial and anticancer activities were not directly correlated. Extracts with strong antibacterial activity, such as K12 and K22, showed proliferative effects in anticancer assays, whereas K10 displayed weak antibacterial but strong anticancer activity. These findings demonstrate that antibacterial and anticancer properties of propolis are governed by distinct phytochemical compositions. Overall, this study highlights the multidimensional bioactivity of Turkish propolis and emphasizes the importance of comprehensive phytochemical characterization for identifying extracts with application-specific therapeutic potential.

## 1. Introduction

The “One World–One Health” concept was developed in 2004 and is based on the tight interaction between human health and that of other organisms (e.g., animals, infectious agents) and the shared environment. Recently, cancer has been proposed to be evaluated within the One Health concept [1], emphasizing that cancer development and progression are influenced not only by genetic and physiological factors but also by environmental exposures, ecosystem dynamics, and microbial interactions. Similarly, antimicrobial resistance (AMR) has emerged as one of the most critical global health threats, further emphasizing the urgent need for novel bioactive compounds with therapeutic potential under the One Health approach [2].

Honeybees (*Apis mellifera*) are strong players in “One World–One Health” owing to their crucial roles in ecosystems, with their pollinator roles providing stability in the ecosystem and food sustainability [3]; this may also meaningfully support the claim “*One Health*, *One Hive*”. Honeybee products including honey and propolis have benefits on human health besides their nutritional effects [4]. Among the bee products, propolis is important with its high load of bioactive components. Propolis is a generic name produced from the Greek words *pro-* (before) and *-polis* (city) and acts as the protective substance of bee hives. Its rich chemical composition has made propolis a valuable commodity throughout history; its utilization as a medicine or health supplement dates back to thousands of years ago, and it can be found in folk medicine worldwide, including Egyptian, Chinese and Greek medicine [5]. In the 17th century, propolis was prescribed as antimicrobial medicine in England and showed popularity in Europe until the 20th century. In addition, it was used for the treatment of tuberculosis in the Soviet Clinics during World War II [6]. The health benefits of propolis are related to the origin of propolis that determines chemical and pollen compositions, which are dictated by the flora [7]. Health benefits of propolis have been widely studied during the last three decades for antimicrobial, anticancer [8], anti-aging [9], anti-obesity [10] and anti-hair loss [11] benefits. However, the contribution of individual phytochemicals and their synergistic interactions remains incompletely understood. The diverse bioactive compounds in propolis have direct relation with the biological effects, and there is a pattern for abundancy of these bioactive compounds in propolis depending on the geographical region. Phenolic acid esters and flavonoids are abundant in the propolis samples from temperate regions (e.g., Europe) compared to that of tropical region propolis samples, which contain high amounts of *p*-coumaric acid derivatives, flavonoids and terpenes [7].

Although numerous studies have examined the antioxidant, antimicrobial, and anticancer properties of propolis independently, comprehensive studies integrating phytochemical characterization with multiple biological activities remain limited, particularly for Turkish propolis samples collected from diverse geographical regions. These studies reveal that Turkish propolis contains high amounts of phenolic compounds and has strong antioxidant effects. Thanks to the dense forested areas in Türkiye, propolis has a wide floral source and diversity. This diversity, along with the different uses of propolis produced in the region, can be explored. Therefore, studies conducted in different geographical areas can contribute to revealing the types and properties of propolis. Moreover, the relationship between chemical composition and different biological activities is still not fully elucidated. Therefore, the aim of the present study was to comprehensively evaluate thirty Turkish propolis extracts collected from different geographical regions by investigating their phytochemical composition, antioxidant capacity, antibacterial activity, and anticancer potential. In addition, Pearson correlation analyses were performed to identify the relationships between phytochemical composition and biological activities. This integrated approach provides a better understanding of the chemical determinants underlying the diverse biological activities of Turkish propolis in Kastamonu Province and may contribute to the identification of region-specific propolis samples with application-oriented therapeutic potential.

## 2. Results and Discussion

### 2.1. Palynological Characterisctics of the Extracts

**Figure 1:**
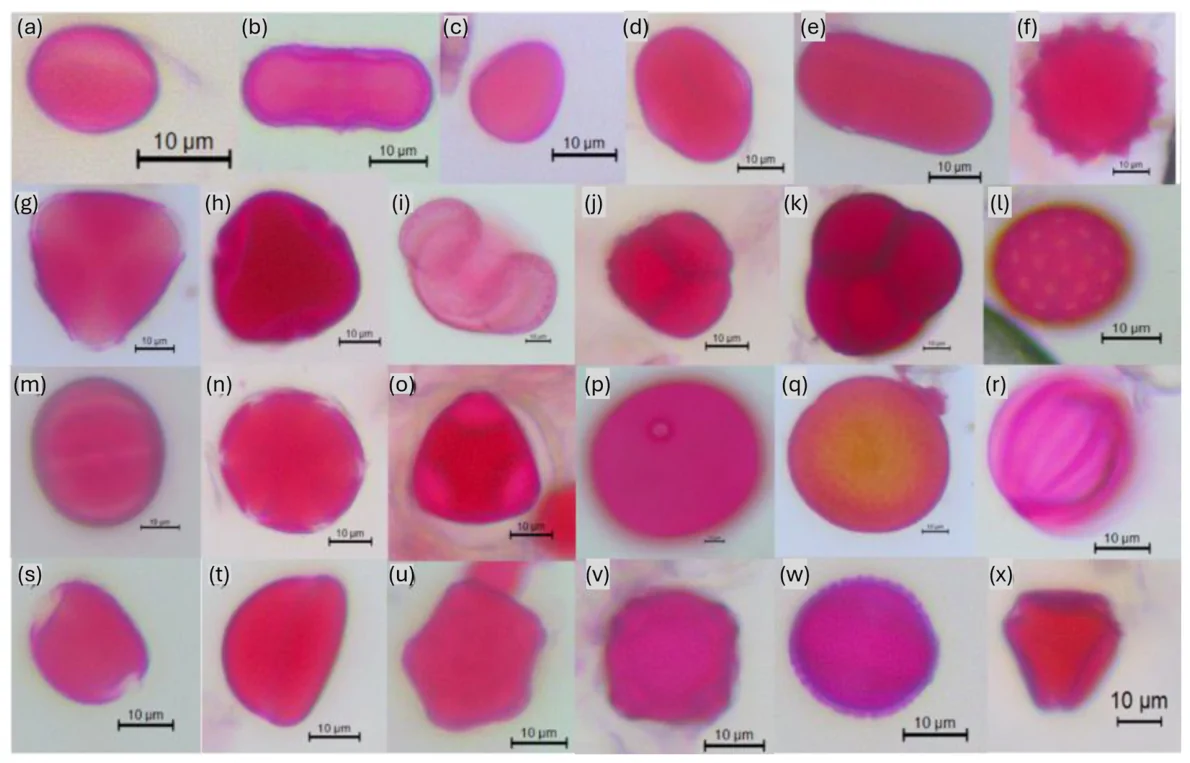
Light microscope images of pollen grains ((**a**) *Castanea* sp., (**b**) *Daucus* sp., (**c**) *Echium* sp., (**d**) *Trifolium* sp., (**e**) *Onobrychis* sp., (**f**) Asteraceae, (**g**) Rosacea, (**h**) *Tilia* sp., (**i**) Pinaceae, (**j**) *Erica* sp., (**k**) *Rhododendron* sp., (**l**) *Chenopodium* sp., (**m**) Lamiaceae, (**n**) Lamiaceae, (**o**) *Morus* sp., (**p**) Poaceae, (**q**) *Cornus* sp., (**r**) *Polygala* sp., (**s**) *Muscari* sp., (**t**) *Allium* sp., (**u**) *Ulmus* sp., (**v**) *Campanula* sp., (**w**) *Hedera* sp., (**x**) *Myrtus* sp.).

The palynological analysis (Figure 1) showed that the propolis samples contain pollen from the Amaranthaceae, Amaryllidaceae, Apiaceae, Araliaceae, Asparagaceae, Asteraceae, Boraginaceae, Brassicaceae, Caryophyllaceae, Campanulaceae, Cistaceae, Cornaceae, Ericaceae, Fabaceae, Fagaceae, Lamiaceae, Myrtaceae, Malvaceae, Moraceae, Papaveraceae, Polygalaceae, Pinaceae, Poaceae, Ranunculaceae, Rosaceae, Salicaceae, and Ulmaceae families, totaling 27 families and 37 genera.

Palynological analysis revealed substantial floral diversity among the Turkish propolis samples, which is a clear sign of multifloral botanical origin with considerable regional variability. Among the identified plant families, Fabaceae and Fagaceae were identified as the most dominant pollen sources, appearing as dominant (D) or secondary (S) taxa in a large proportion of the samples (Table S1). Rosaceae also showed a significant contribution, being a dominant or secondary pollen source in several propolis samples. In contrast, plant families such as Apiaceae, Araliaceae, Asteraceae, Boraginaceae, and Malvaceae were mainly detected at minor (M) levels, while Amaranthaceae, Amaryllidaceae, Campanulaceae, Salicaceae, and Ulmaceae were generally present in trace amounts (T).

This distribution suggests that resin collection by honeybees is influenced by both dominant woody vegetation and surrounding herbaceous flora. Palynological studies have revealed that the taxa identified are largely consistent with the local flora.

The prevalence of Fabaceae–Fagaceae–Rosaceae as major pollen contributors indicates the presence of shared botanical sources across different ecological regions of Kastamonu Province.

### 2.2. Chemical Composition of the Extracts

Chemical composition analysis revealed that there was a significant variation observed in the phytochemical composition and antioxidant capacity of the 30 Turkish propolis extracts, highlighting the strong influence of geographical origin on chemical composition. Total flavonoid content (TFC) ranged from 135.57 to 1101.91 mg QE/g, while total phenolic content (TPC) varied between 7.71 and 191.27 mg GAE/g. The highest TFC values were obtained for K22 (1101.91 mg QE/g), K24 (1095.95 mg QE/g), and K12 (1030.39 mg QE/g), indicating flavonoid-rich extracts. Similarly, K12 exhibited the highest phenolic content (191.27 mg GAE/g), followed by K24 (165.59 mg GAE/g) and K13 (144.11 mg GAE/g). Antioxidant assays further showed that there were marked differences among the extracts; FRAP values ranged from 7.56 to 119.40 mg AA/g, with K12 showing the strongest ferric reducing antioxidant power, followed by K24 and K13. DPPH radical scavenging activity also varied substantially, ranging from 11.32% to 87.80%. K15 exhibited the highest DPPH scavenging activity, while K30 showed significantly low antioxidant activity across all the tested parameters. These findings clearly demonstrate substantial compositional and functional heterogeneity among the tested Turkish propolis samples.

**Figure 2:**
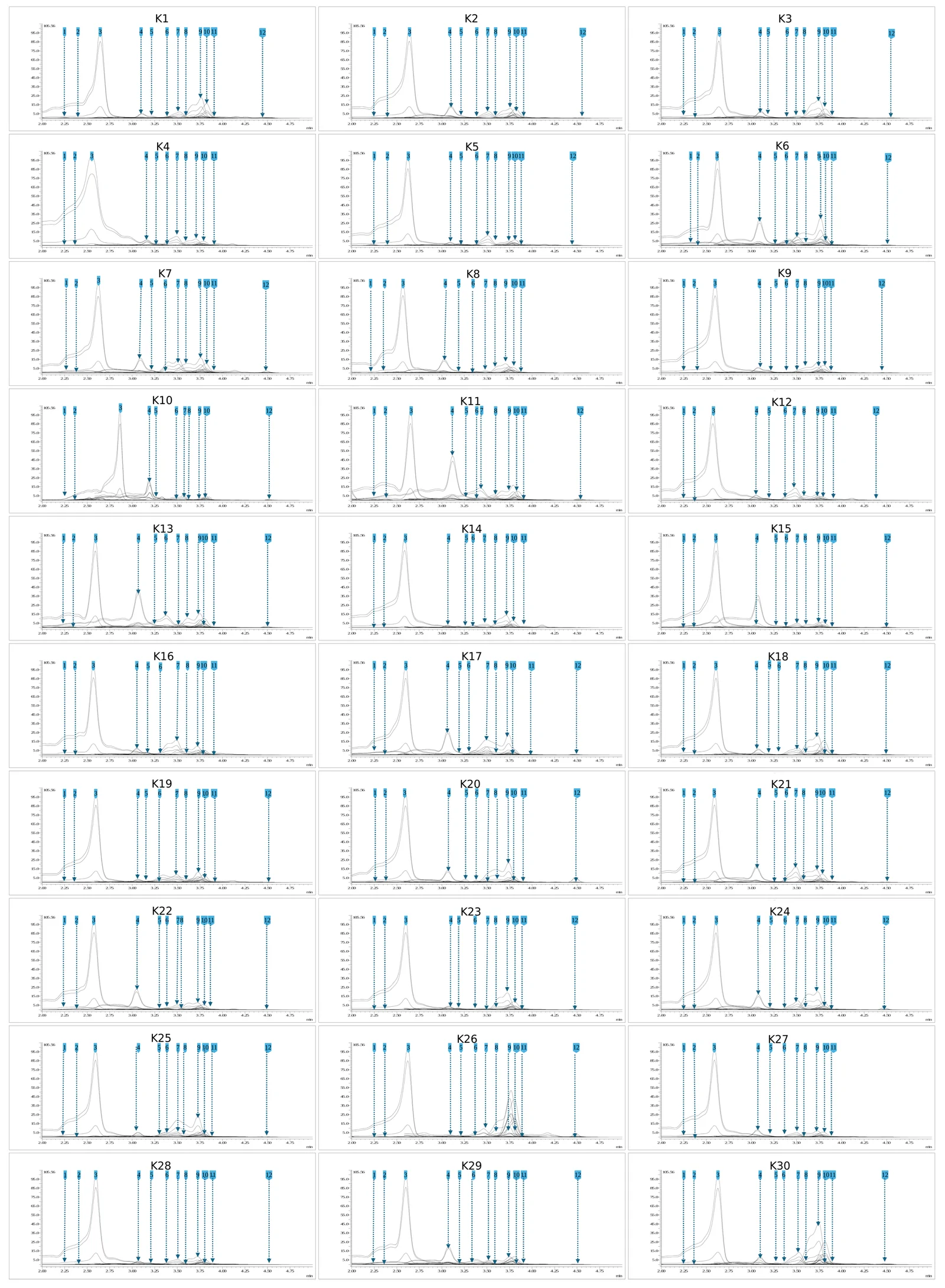
LC chromatograms (1: Catechin, 2: 2-5 dihydroxy benzoic acid, 3: Caffeic acid, 4: Trans-ferulic acid, 5: Rutin trihidrat, 6: Myricetin, 7: Cinnamic acid, 8: Ellagic acid, 9: Quercetin, 10: Naringenin, 11: Luteolin, and 12: Chrysin).

Detailed phenolic profiling revealed variations in the abundance of major phenolic compounds among the extracts, suggesting region-dependent phytochemical signatures. Among the quantified compounds, caffeic acid, quercetin, cinnaminic acid, and trans-ferulic acid emerged as the predominant phenolics in most samples, while CAPE was detected at lower but relatively consistent levels. Caffeic acid showed particularly high abundance in several extracts, including K4 (172.97), K12 (91.63), and K14 (81.54), whereas quercetin reached its highest concentration in K25 (9.145) and K13 (8.519). Trans-ferulic acid was also abundant in selected samples, especially K22 (34.15) and K21 (25.57). In terms of cinnaminic acid, K16 (50.36), K12 (34.71), and K4 (34.05) showed the highest concentrations, while K20 (1.06) had the lowest concentration (Table 1; Figure 2). The predominance of phenolic acids and flavonoids is consistent with the characteristic composition of temperate-region propolis, which is known to be rich in phenolic acid esters and flavonoids [7]. The observed phytochemical diversity likely explains the differences in antioxidant and biological activities among the extracts. In particular, extracts with high flavonoid richness and elevated concentrations of quercetin and ferulic acid generally exhibited stronger antioxidant performance [12,13], suggesting that both total abundance and qualitative composition of phenolics contribute to the bioactivity of Turkish propolis.

The spatial distribution maps of ellagic acid, quercetin, apigenin, and triacetin show an increase, particularly in the southwestern regions of the province. TPC and FRAP values, on the other hand, are relatively higher in samples collected from the western part of the province. However, many studies have reported that the highest TPC levels are found in propolis derived from willow and poplar trees in deciduous forested areas [14,15].

**Figure 3:**
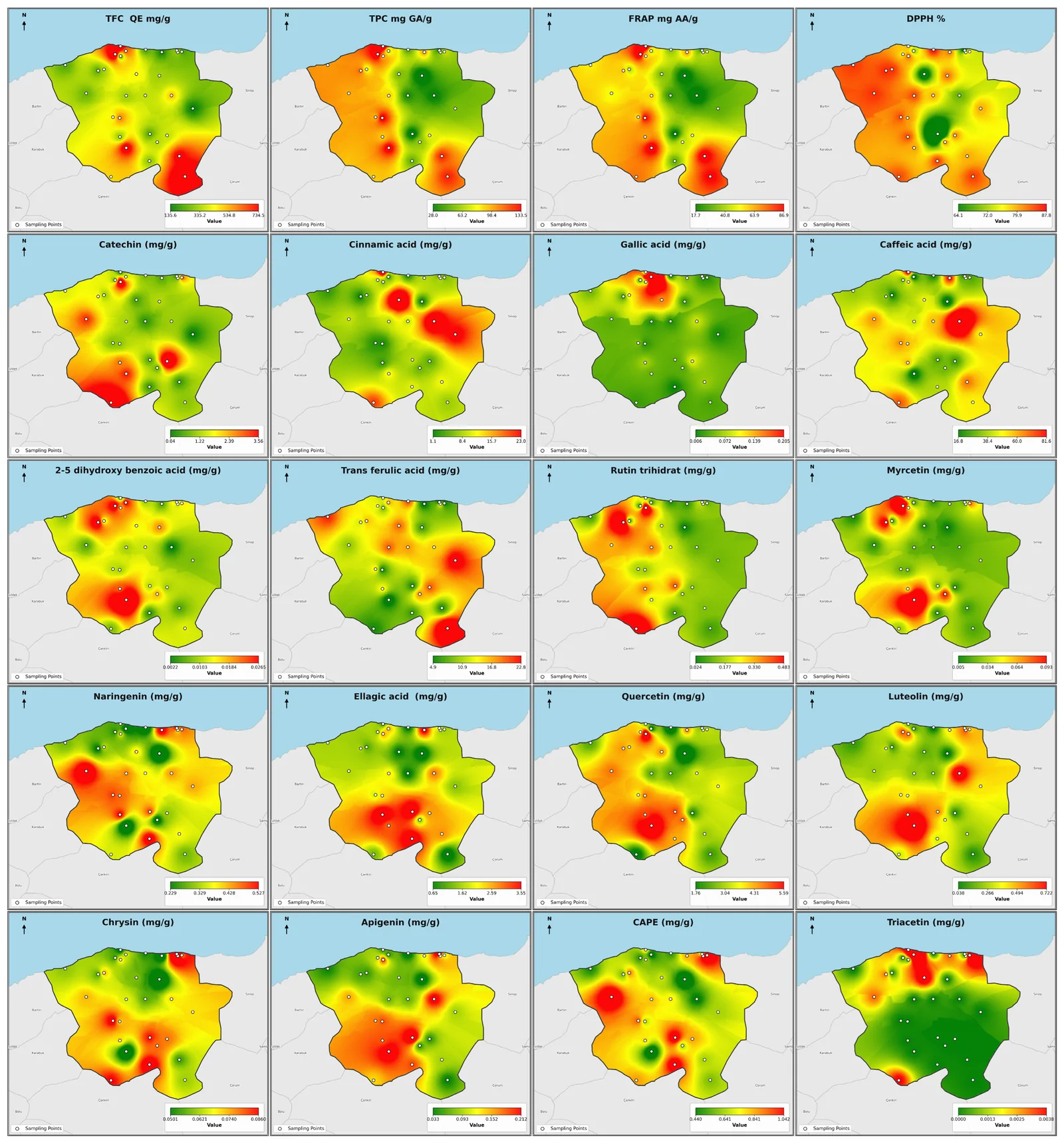
Spatial distribution maps of the chemical compounds and antioxidant activity parameters in the extracts.

Spatial distribution maps revealed substantial geographical variation in the phytochemical composition and antioxidant properties of the extracts (Figure 3). Total phenolic content (TPC), total flavonoid content (TFC), and FRAP values exhibited common spatial distribution patterns, with clear hotspots observed in specific regions of Kastamonu Province. In general, western, northwestern, and southwestern regions showed higher TPC, TFC, and FRAP values, revealing the presence of phenolic-rich propolis samples with higher reducing capacity. The similar spatial distribution of these parameters may support the strong association observed between flavonoid content and antioxidant capacity. In contrast, DPPH radical scavenging activity showed a partially distinct distribution pattern, which might be a sign that antioxidant activity among propolis samples may involve multiple underlying mechanisms rather than a single redox-based process [16].

The spatial distribution of individual phenolic compounds further clarified the regional heterogeneity in propolis composition. It has been reported that beech and fir forests have a wide distribution in the southwestern part of the province and in areas of similar altitudes. This situation may contribute to explaining the differences in the amounts of ellagic acid, quercetin, apigenin, and triacetin. CAPE and chrysin, however, showed largely similar distributions. Ristivo-jević et al. reported galangin, CAPE, chrysin, and pinocembrin as marker compounds for poplar-type propolis. Although the Salicaceae taxon was not identified as dominant in palynological analyses, it was generally detected in these samples. This supports the idea that palynological analyses are a more suitable tool for revealing the regional flora rather than directly determining the botanical origin of propolis [17]. Among the quantified compounds, caffeic acid exhibited one of the most noticeable geographical variations, with distinct hotspots in specific sampling regions that can be ascribed to major botanical influence on its abundance. Similar differences were observed for quercetin and trans-ferulic acid. These observations are particularly relevant given the correlation between specific phenolic compounds and antibacterial activity observed in this study as described below. The regional phenomenon of phenolic-rich propolis samples indicates that geographical origin is a major determinant of chemical composition.

The qualitative properties of propolis extracts can vary significantly depending on the extraction techniques and duration. This is a limiting factor in comparing the findings with different studies. Similarly, the inadequacy of palynological analyses in identifying the botanical sources of propolis makes it difficult to establish relationships between different studies. Nevertheless, various extracts of propolis can pave the way for the development of products targeting different objectives.

**Figure 4:**
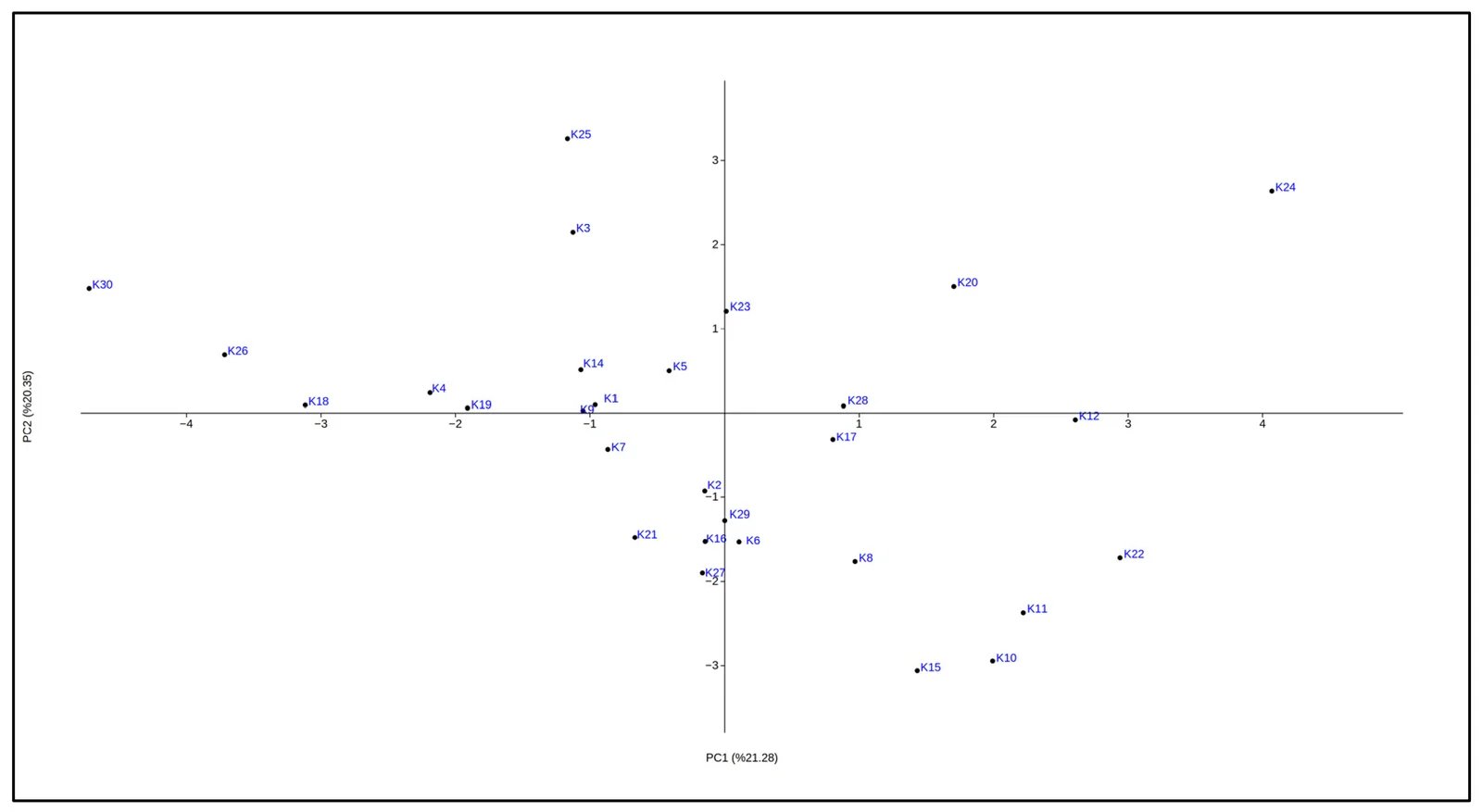
PCA graph prepared according to the active components of propolis samples.

The PCA graph showed a largely homogeneous distribution of the samples. However, samples coded K10, K11, K15, and K22 clustered in region IV, while samples coded K13, K20, and K24 clustered in region I (Figure 4). In the first component of PCA, the strongest distinguishing parameter was the amount of TFC, while in the second component, the strongest parameters were identified as luteolin, quercetin, myricetin, and apigenin. The highest amount of luteolin in sample K13 contributed to its placement at a distant point from the others, while the highest amounts of myricetin were detected in samples coded K13, K20, and K24, which were grouped closely together. The lowest amounts of apigenin were detected in samples coded K10, K11, K15, and K22. However, PCA did not contribute to the geographical separation of the propolis samples examined.

### 2.3. Biological Characteristics of the Extracts

#### 2.3.1. Antibacterial Characterization

The antibacterial activity of 30 geographically distinct propolis extracts (K1–K30) was evaluated against three Gram-positive bacteria (*Staphylococcus aureus*, *Enterococcus casseliflavus*, and *Enterococcus faecalis*) as well as seven Gram-negative bacteria, including *Pseudomonas fluorescens*, *Pseudomonas aeruginosa*, *Enterobacter hormaechei*, *Salmonella enterica* serovar Kentucky, *Enterobacter aerogenes*, *Salmonella enterica* serovar Typhimurium, and *Salmonella enterica* serovar Infantis. Antibacterial efficacy was assessed using inhibition zone diameter (ZI), minimum inhibitory concentration (MIC), and minimum bactericidal concentration (MBC) assays. Significant differences in antibacterial activity were observed among the extracts, indicating substantial differences in antimicrobial potency depending on both extract origin and target microorganism (Table S1 and Table 2).

Among all the tested strains, *S. aureus* was determined as the most susceptible bacterial species, for which inhibition zones ranged from 6.5 to 18.5 mm, with the strongest activities observed for K22 (18.5 mm), K13 (18 mm), and K21 (17.5 mm). The MIC values ranged from 0.08 to 0.63 mg/mL, with most extracts giving MIC values of 0.31 mg/mL, confirming strong anti-staphylococcal activity.

Moderate antibacterial activity was observed against *E. casseliflavus*, with inhibition zones ranging from 7 to 12 mm and MIC values between 0.16 and 0.63 mg/mL. In contrast, *E. faecalis* exhibited greater variability in susceptibility, as several extracts demonstrated high antibacterial activity; the lowest MIC value (0.02 mg/mL) was recorded for K7, K12, K17, K23, K24, and K25.

MIC and MBC tests were performed for the bacterial species with the extracts that caused zone inhibition. For each extract, 2.5 mg/mL was used as the highest concentration since the most vulnerable species showed no growth (bactericidal concentration) at this concentration. K8, K9, K22, K24, and K30 did not cause an MBC effect for *S. aureus* species, while K12 and K30 (which gave ZI) showed no MBC for *E. casseliflavus*. In contrast to these, none of the extracts gave MBC for *P. fluorescens*. *P. aeruginosa*, *E. hormaechei*, *S. kentucky*, *E. aerogenes*, *S. typhimuirum* and *S. infantis* showed relatively higher resistance to the extracts. *E. hormaechei* was the most stubborn species and was only affected by K15 and K30 extracts.

The Gram-negative species generally showed lower susceptibility, including *P. fluorescens*, for which inhibition zones ranged from 8.5 to 15 mm, with K25 showing the highest activity (15 mm). MIC values ranged from 0.02 to 0.63 mg/mL; however, bactericidal activity was largely absent, with only K25 exhibiting measurable MBC at the tested concentrations. In contrast, only selected extracts demonstrated antibacterial activity against *P. aeruginosa*, for which the inhibition zones ranged from 6.5 to 11 mm and MIC values ranged between 0.15625 and 0.625 mg/mL. Notably, all active extracts showed bactericidal activity at an MBC of 2.5 mg/mL. Similarly, antibacterial activity against *E. hormaechei*, *S. Kentucky*, *E. aerogenes*, *S. Typhimurium*, and *S. infantis* was extract-dependent and generally low to moderate. Among these, K12 showed particularly strong activity against *E. aerogenes*, for which the lowest MIC value (0.078125 mg/mL) was obtained. It is worth noting that the antibacterial activity against *E. faecalis* was notably more heterogeneous, which may be attributed to the susceptibility depending on the specific phytochemical profile of each extract. This variability may also indicate selective activity of certain propolis-derived compounds against enterococcal targets. The extracts K12, K15, K16, K21, K25, K27, K28, and K30 demonstrated the broadest antibacterial spectrum for both Gram-positive and Gram-negative bacterial strains. Among all tested extracts, K25 was obtained as the most promising candidate due to its broad-spectrum activity and consistently strong antibacterial performance across multiple strains.

Consistent with previous studies, Gram-positive bacteria were generally more susceptible to propolis extracts than Gram-negative bacteria [18]. This enhanced susceptibility is commonly attributed to the structural characteristics of Gram-positive bacteria, whose exposed peptidoglycan layer facilitates interaction with bioactive compounds such as flavonoids, phenolic acids, and terpenoids [19]. However, several studies have reported that propolis is also effective against Gram-negative bacteria [20,21]. The strongest antibacterial activity was observed against *S. aureus*, which may be related to the fact that this species is particularly sensitive to propolis-derived antimicrobial compounds. As expected, Gram-negative bacterial species have greater resistance in general, which can largely be explained by the presence of an outer membrane acting as a permeability barrier. Nevertheless, several extracts showed remarkable inhibitory activity against clinically and microbiologically relevant Gram-negative genera, including *Pseudomonas*, *Enterobacter*, and *Salmonella*. Taken together, these findings support the potential application of region-specific propolis extracts as natural antimicrobial agents. Future studies should focus on detailed phytochemical characterization and multivariate correlation analyses to identify the compounds responsible for antibacterial activity and clarify the mechanisms underlying strain-dependent susceptibility.

However, various studies report that propolis extraction methods directly affect its antimicrobial potency. Veronica et al. reported that glycerin-based propolis extracts were more effective than ethanolic and aqueous extracts. Similarly, a study on Brazilian propolis reported that ethanol was more effective than water without dissolving the active compounds [22,23].

#### 2.3.2. Influence of Phenolic Composition on Antibacterial Activity

The detailed phenolic profiling (LC-MS/MS) further revealed that antibacterial activity was more strongly associated with specific phenolic compounds, rather than with total flavonoid content alone. Even though certain extracts gave exceptionally high total flavonoid content, the antibacterial capacity did not correlate with total flavonoid abundance, showing the importance of qualitative rather than purely quantitative compositional differences. Among the identified phenolic compounds, caffeic acid emerged as one of the most prominent candidates associated with the obtained antibacterial activity. The extracts possessing strong and broad-spectrum antibacterial activity, including K12, K23, K25, and K28, generally contained high amounts of caffeic acid. This observation is consistent with previous studies revealing that caffeic acid contributes to antibacterial activity through membrane destabilization, oxidative stress induction, and disruption of bacterial energy metabolism [24]. Similarly, quercetin was determined another major contributor to the observed antibacterial activity. The extracts (e.g., K13 and K25) containing relatively higher quercetin levels strong antibacterial activity. It is known that Quercetin has been reported to inhibit bacterial growth by altering membrane permeability, suppressing nucleic acid synthesis, and interfering with biofilm formation [25,26].

Although cinnamic acid and trans-ferulic acid were also abundant in several extracts, their contributions appeared to be more species-dependent. In particular, trans-ferulic acid may be effective in enhancing the antibacterial activity against Gram-negative bacteria and could potentially facilitate interactions with outer membrane components. Interestingly, CAPE (caffeic acid phenethyl ester), despite being a well-recognized bioactive marker of propolis [27], did not show a clear direct correlation with antibacterial activity under the test conditions.

Pearson correlation analysis showed the distinct associations between phytochemical parameters and the obtained antibacterial activity. Among all evaluated variables, FRAP showed the strongest positive correlation with overall antibacterial activity (r = 0.498), depicting that higher reducing antioxidant capacity is linked to antimicrobial efficacy. In addition, total flavonoid content (TFC) also gave a moderate positive correlation (r = 0.467), which can be ascribed to the tendency of flavonoid-rich extracts to possess stronger antibacterial activity. Interestingly, total phenolic content (TPC) showed only a weak correlation with the antibacterial performance (r = 0.217), supporting the idea that antibacterial efficacy is not determined solely by total phenolic abundance, while the qualitative composition of phenolic constituents seems to be more relevant.

Bacteria-specific correlation analysis revealed differential patterns among the bacterial species. For example, the obtained antibacterial activity against *S. aureus* showed the strongest correlations with DPPH, FRAP, and TFC, whereas the antibacterial activity against *P. fluorescens* was more closely associated with ferulic acid and antioxidant capacity. These findings suggest that different bacterial species may be affected by distinct groups of bioactive compounds within the extracts. Overall, the correlation analysis supports the hypothesis that antibacterial activity in propolis extracts is primarily driven by antioxidant capacity and specific phenolic composition rather than total phenolic content alone [18].

Comparing the phytochemical and biological activity data, the palynological findings suggest that floral composition plays a critical role in determining the chemical profile and functional properties of propolis. The extracts containing pollen grains in Fabaceae, Fagaceae, and Rosaceae generally showed higher phenolic and flavonoid contents as well as stronger antioxidant capacity. This may be a sign that these botanical sources are the main contributors to the presence of bioactive compounds. Similarly, the propolis samples showing stronger antibacterial activity tended to contain more complex or richer pollen profiles, suggesting that biological efficacy may depend not only on total phenolic abundance but also on floral diversity and botanical origin. These findings support the hypothesis that geographical and floristic variation are major determinants of the phytochemical heterogeneity observed in Turkish propolis.

#### 2.3.3. Anticancer Characteristics of the Extracts

The anticancer activity of propolis extracts showed varied and time-dependent effects on cell viability, revealing both cytotoxic and proliferation supportive responses depending on the extract composition and exposure duration. Several extracts showed strong and continued cytotoxic activity. Among them, K10 showed the most promising inhibitory effect, with inhibition rates of 80.25%, 85%, and 65.75% at 24, 48, and 72 h, respectively. This shows potent and persistent anticancer activity. Similarly, K4 displayed strong inhibition across all the tested time periods (the inhibition rates of 52.5%, 42.5%, and 30.75%), for which the cytotoxicity was sustained but gradually declined. K23 also showed significant cytotoxicity, particularly at 24 and 72 h, with inhibition rates of 40.25% and 50.25%, respectively. Some extracts showed time-dependent enhancement of cytotoxicity. For example, K3 exhibited progressively increasing inhibition as the inhibition rose from 11.75% at 24 h to 38.25% at 72 h, which can be related to cumulative anticancer effects. Likewise, K7 showed minimal effect at 24 h, followed by increased inhibition at 48 and 72 h (14.5% and 35.5%, respectively). K21 also demonstrated a time-dependent inhibitory response as an increase from 0.5% at 24 h to 32% at 72 h.

Some other extracts displayed transient cytotoxicity followed by recovery in cell viability. For example, K1 showed strong early inhibition (51% at 24 h), which declined at later time points and disappeared at 72 h, indicating loss of cytotoxic activity over time. Similarly, K6 showed moderate inhibition at 24 and 48 h (24% and 40.5%) but nearly lost its cytotoxic activity at 72 h. Another example is that K14 followed a comparable trend, as the inhibition decreased dramatically from 47.75% at 24 h to 3.25% at 72 h.

In contrast, the extracts K5, K9, K11, K12, K13, K15, K17, K19, and K22 stimulated cell proliferation rather than possessing any cytotoxicity. Particularly, K12 and K22 exhibited strong proliferative effects, while K15 provided the highest increase in viability (111% at 72 h). A biphasic response was observed in certain extracts. For example, K20 showed an antiproliferative effect at 24 and 72 h but a slight proliferative effect at 48 h, while K8 displayed mild inhibition at 24 and 72 h but increased viability at 48 h. Based on these findings, it can be concluded that propolis extracts may exert concentration- and time-dependent dual cytotoxic and proliferative effects depending on the floral origin.

It is worth mentioning that the proliferative effects observed for several propolis extract treatments should be interpreted cautiously. Since extracts were dissolved in an aqueous ethyl alcohol, it is possible that certain propolis samples exerted cytoprotective effects against solvent-induced cellular stress rather than directly stimulating proliferation. It is known that the antioxidant and membrane-protective properties of propolis-derived phenolics might improve the cell survival [28,29] by mitigating oxidative or metabolic stress caused by the vehicle system.

Interestingly, comparison of antibacterial and anticancer results revealed that there was no direct relationship between the antibacterial activity and the anticancer potential of the propolis extracts. Several extracts possessing relatively high TPC, TFC, and FRAP values (e.g., K12 and K22) showed broad-spectrum antibacterial activity against both Gram-positive and Gram-negative bacteria, while they played proliferation-supportive roles in anticancer assays, for which increased cell viability relative to the control at all evaluated time points was obtained. Conversely, K10, which exhibited comparatively weak antibacterial activity and low phytochemical richness, demonstrated the strongest anticancer activity. Similar results were obtained for the extract K4 with moderate antibacterial activity and strong cytotoxic effects against cancer cells. These findings may be ascribed to the fact that antibacterial and anticancer activities are governed by distinct bioactive mechanisms and may be influenced by different phytochemical compounds/compositions. While antibacterial activity appeared to correlate more strongly with antioxidant capacity and flavonoid richness, the observed anticancer activity showed a more complex and less predictable pattern.

## 3. Materials and Methods

### 3.1. Field of Study

Kastamonu Province, chosen as the study area, has a rugged terrain ranging from sea level to an altitude of 2565 m. The Küre Mountains, located north of the provincial center and running parallel to the Black Sea coast, and the Ilgaz Mountains to the south, form a transition zone between maritime and continental climates. Flora studies report the areas hosting over 1800 taxa, 14% of which are endemic. Broadleaf and coniferous mixed forests cover 70.71% of the province, agricultural areas cover 28.68%, and settlements cover 0.47%. In the forested areas along the coast, species such as Scots pine, Aleppo pine, black pine, fir, yew, and deciduous species such as beech, oak, ash, maple, alder, elm, chestnut, linden, boxwood, wild hazelnut, poplar, hornbeam, and plane tree are found, while beech and fir trees become more common as one moves inland from the coast. In the forests of Tosya, located in the southernmost part of the province, species such as black pine, Aleppo pine, oak, and fir are encountered. There are 1150 officially registered beekeeping businesses/individuals in the province, with 77,508 beehives. However, the amount of honey produced per hive is 4 kg, which is considerably below the national average (12.5 kg) [30] (TEPGE, 2025). MCF-7 cells (MCF7, (HTB-22), ATTC) were maintained in Dulbecco’s Modified Eagle’s Medium/Ham’s Nutrient Mixture F12 (DMEM/F-12, Gibco, Thermo Fisher Scientific, Waltham, MA, USA) medium supplemented with 10% heat-inactivated fetal bovine serum (FBS, Gibco), L-glutamine, and penicillin-streptomycin (Gibco). 3-(4,5-Dimethylthiazol-2-yl)-2,5-diphenyltetrazolium bromide (MTT) was purchased from Gibco.

### 3.2. Collection of the Propolis Samples

**Figure 5:**
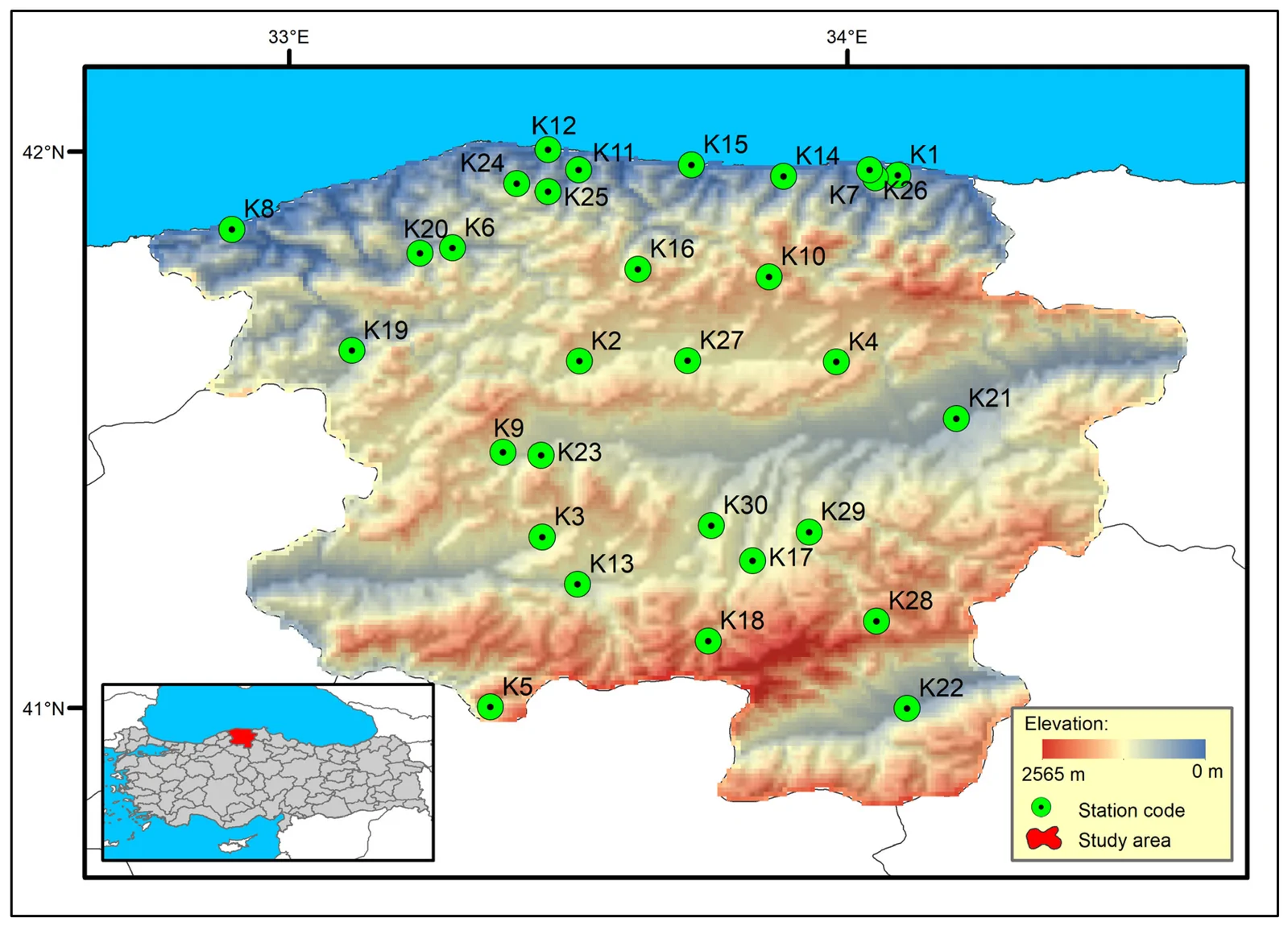
Bee propolis collection locations.

The bee propolis samples used in this study were obtained from the producers operating in Kastamonu Province with the help of the Kastamonu Beekeepers Association. Considering that a worker bee can travel an average distance of four to five kilometers, the apiaries from which the samples were collected were at least five kilometers away from each other. In addition, any propolis samples from the migratory beekeeping were not included into the study. Twenty-two of the collected samples were from the 2024 harvest, and eight were from the 2023 harvest (Figure 5). All 30 samples obtained were collected using the hive scraping method. Propolis samples were stored at −20 °C until extraction and analysis.

### 3.3. Morphological Characterization of Pollen Samples

The method applied by Warakomska and Maciejewicz (1992) for the detection of pollen in propolis samples was modified and applied [31]. According to this method, the pollen preparation process was carried out as follows: (i) a 0.5 g sample of propolis was ground using a blender and transferred to a sterile falcon tube, where (ii) 10 mL of a mixture of ether, acetone, and ethanol in a 1:1:1 ratio was added to the propolis sample, (iii) followed by addition of one mL of acetic acid. The mixture was vortexed for several minutes to create a homogeneous solution, which was then filtered through a sieve (0.25 mm pore size). After the filtration process, the mixture was centrifuged at 4000 rpm for 20 min, and the supernatant was discarded. The precipitate was mixed with 5 mL of the ether, acetone, and ethanol mixture and 1 mL of acetic acid once again, and then the mixture was vortexed for a few minutes.

The homogenized sample was re-filtered through a 250 µm sieve. After filtration, the mixture was centrifuged at 4000 rpm for 20 min. After the centrifugation process was completed, the supernatant was carefully removed. Then, approximately 1–2 mm^3^ of basic fuchsin-glycerin gelatin was taken with a sterile needle, applied to the pellet formed in the centrifuge tube, and transferred to a slide. The slide was heated to 30–40 °C to melt the basic fuchsin-containing glycerin gelatin, and a 18 × 18 mm coverslip was placed on top. The prepared specimen was inverted and left to dry and was ready for examination after approximately 12 h. Objectives with 40× and 100× magnification were used to identify the pollen. Pollen counts were performed using the 40× objective.

### 3.4. Chemical Composition Characteristics of the Samples

Two grams of fine powdered propolis samples (pre-treated for removal of wax and chunks) were placed in a sterile Falcon tube, to which 10 mL of 70% ethyl alcohol was added. The samples were then placed on an orbital shaker for 24 h (150 rpm). The dissolved propolis was centrifuged at 4000 rpm for ten minutes, and the supernatant was filtered through Whatman A4 filter paper. The collected extracts were stored in a refrigerator at +4 °C until use.

*Total Phenolic Content*: The most commonly used method for determining the amount of phenolic compounds is the Folin–Ciocalteu (F-C) method, which was used to determine the total phenolic content as described in the literature [32,33]. The mixture of 200 µL of extract (2–5 times diluted from the stock) was mixed with 200 µL of F-C reagent, which was vortexed and incubated for 5 min. After the incubation, 2 mL of 7% Na_2_CO_3_ and 2.6 mL of *d*H_2_O were sequentially added to the extract/F-C reagent mixture, which underwent thorough mixing and incubation in the dark for 90 min. At the end of incubation, the samples were centrifuged for 5 min at 4000 *g* and the absorbance was read at 760 nm using a UV spectrophotometer [34]. The analysis and measurements were repeated three times for each sample. The obtained data are presented as mg GAE/g propolis extract equivalent.

*Total Flavonoid Content*: The method used by Zhishen et al. (1999) [35] for the total flavonoid content analysis of propolis extracts was modified and used. The basic principle of this method is based on the ability of AlCl_3_ to form stable complexes with the C_4_ keto group, C_3_ or C_5_ hydroxyl groups of flavon, and flavonol components in an acidic environment. In addition, AlCl_3_ can also form complexes with the orthodihydroxyl groups in the A and B rings of flavonoids. It was determined that the absorbance values obtained were directly proportional to the flavonoid content. Quercetin was used as a standard to determine the total flavonoid content. The extracts and the reactants were sequentially added into 15 mL polypropylene centrifuge tubes: 300 μL of propolis extract (2–10 times diluted from the stocks), 3.4 mL of 30% methyl alcohol, 150 µL of 0.5 M NaNO_2_ and 150 µL of 0.3 M AlCl_3_·6(H_2_O). The mixtures were vortexed and incubated for 5 min at room temperature, in which 1 mL of 1M NaOH was added right after incubation. Absorbance values of the samples were determined at 506 nm using a UV spectrophotometer. Analyses and measurements were repeated three times for each sample [36]. The results were presented as mg Quercetin/g propolis extract equivalent.

*Ferric Fe^3+^ Reducing Power Assay*: A total of 500 μL of the extract was diluted with 500 μL of ethanol, and 2.5 mL of phosphate buffer (pH = 6.6) was added to the mixture. Then, 2.5 mL of a 1% K_3_Fe(CN)_6_ solution was added to the mixture, and it was incubated at 50 °C for 20 min. After incubation, 2.5 mL of a 10% TCA solution was added, and the mixture was centrifuged at 3000 rpm for 10 min. From the upper phase, 2.5 mL was taken, to which 2.5 mL of *d*H_2_O and 500 μL of FeCl_3_·6H_2_O were added; after 2 min incubation, a measurement was taken at a wavelength of 700 nm [37].

*DPPH Radical Scavenging Activity Method*: The free radical scavenging capacity of propolis was determined using the DPPH method. For this purpose, 500 µL of propolis extract prepared at a concentration of 0.5 mg/mL was mixed with 500 µL of *d*H_2_O and 2 mL of 0.2 mM DPPH, then vortexed and incubated for 20 min. For the control, the same procedure was performed using 1 mL of methanol and 2 mL of DPPH solution. After incubation, the samples were measured at a wavelength of 517 nm, and the percentage inhibition value was calculated [38].

*LC-MS/MS-Based Characterization*: The phenolic composition of propolis extracts was determined using a Shimadzu LCMS-8030 Plus system (Shimadzu Corporation, Kyoto, Japan). The LC-MS/MS technique enables the identification of compounds analyzed by high-pressure liquid chromatography, which separates compounds based on their physicochemical properties using mass spectrometry; this is among the most widely used and trusted techniques used in the analysis of propolis extracts [39,40]. Prior to the analysis, 2 g of propolis sample was dissolved in 20 mL of 70% ethanol and filtered to prepare the extracts. Injections of 10 μL were performed at a flow rate of 0.4 mL/min using an Inertsil ODS4 column (3 μm; 2.1 × 50 mm; GL Sciences Inc., Tokyo, Japan) at a column temperature of 40 °C, with water containing 1‰ formic acid (Mobile Phase A) and methanol containing 1‰ formic acid (Mobile Phase B) as mobile phases. The elution gradient program was set as follows: 5% B (0–4 min), 95% B (4–7 min), and 5% B (7–12 min). The operating conditions were used as described in the literature [39].

### 3.5. Bioactivity of the Propolis Extracts

#### Determination of Antimicrobial Activity

The antimicrobial activity of propolis extracts were performed in combination with zone inhibition, minimum inhibitory concentration (MIC), and minimum bactericidal concentration assays. *The disk diffusion method* is widely used to determine zone inhibition, which is assigned to antimicrobial activity. The antibiogram disks with a diameter of 6 mm containing an antimicrobial agent at a specific concentration (total of 60 µL from 200 mg/mL was slowly impregnated to the paper disks) were carefully placed on the surface of the solid medium, where the microorganism was inoculated using a sterile forceps. A distance of 22 mm was maintained between the disks and 14 mm between the disks and the edge of the Petri dish to prevent the inhibition zones from interacting with each other. The cultures were incubated at 37 °C for 18–24 h, and the diameter of the growth free zone was measured with a caliper. As the microorganism’s sensitivity to the antibiotic increases, the diameter of these inhibition zones widens [41]. As positive controls, Ampicillin (AM) MCG 2; Ceftazidime (CAZ) MCG 10; Gentamicin (CN) MCG 10; Meropenem (MEM) MCG 10; Ceftriaxone (CRO) MCG 30; Tazobactam/Piperacillin (TPZ) MCG 36; and Vancomycin (VA) MCG 5 standard antibiotic disks were used. The negative control was performed using disks impregnated with only the solvent. Minimum inhibitory and minimum bacterisidal assasys were performed in accordance with the literature [42,43]. An amount of 2.5 mg/mL was selected as the highest tested concentration in MIC and MBC tests due to the fact that most propolis samples have MIC values below 2.5 mg/mL for most bacterial species [18].

### 3.6. Anticancer Tests

Anticancer tests were performed as described in the literature [44,45]. MCF-7 cells were cultured in DMEM/F12 supplemented with 10% FBS and penicillin–streptomycin (1%) in 37 °C and a 5% CO_2_ humidified incubator. Viability of MCF-7 cells was assessed by MTT assay. Cells were seeded into 96-well plates at a density of 10^4^ cells/well and treated with the propolis extracts or 70% aqueous ethanol (vehicle control) for 24, 48, and 72 h. At the end of the incubation, the culture medium was discarded, the cells were rinsed with PBS buffer, and fresh DMEM/F12 containing 0.5 mg/mL MTT and 0.5% FBS was added to the cell flask and incubated further for 4 h at 37 °C. The purple formazan crystals were solubilized with DMSO, and the absorbance values at 570 nm were measured with a multi-plate reader. All the experiments were repeated three times.

### 3.7. Statistical Analyses

Pearson correlation analysis was performed to investigate the effects of the physicochemical properties of propolis on its antimicrobial and anticancer activity. PCA was used to examine the contribution of chemical content parameters in distinguishing propolis types. All parameters in Table 1 and Table 3 were normalized using the z-score method and prepared with the PAST v 5.2.1 program.

## 4. Conclusions

This study provided a comprehensive evaluation of Turkish propolis collected from diverse geographical regions within Kastamonu Province by integrating phytochemical profiling, antioxidant capacity, antibacterial activity, and anticancer potential. The results revealed that there was substantial variability among propolis samples in terms of chemical composition and biological activity, confirming that geographical origin is a major determinant of propolis quality and biological functionality. Phenolic-rich samples, particularly those with elevated total phenolic content, total flavonoid content, and strong reducing power, generally exhibited superior antibacterial capacity. Antibacterial analyses revealed stronger activity against Gram-positive bacteria, although some of the extracts showed promising inhibitory effects against Gram-negative strains. Correlation analyses further supported the relationship between phenolic composition, antioxidant properties, and antibacterial efficacy while the preliminary anticancer study did not show any trend or statistically significant outcome with the composition. Overall, these findings highlight Turkish propolis as a highly diverse natural product with considerable therapeutic potential (particularly antibacterial) and emphasize the importance of region-specific characterization for the development of standardized propolis-based functional and pharmaceutical products.

## 5. Limitations of the Study

One limitation of the present study is that the quantitative LC-MS/MS analysis was restricted to a predefined panel of flavonoids and phenolic acids available within the analytical service package used for phytochemical quantification. Consequently, although several major phenolic compounds were quantified, the analysis did not encompass the full spectrum of phenolic acids, flavonoids, and other potentially bioactive phytochemicals that may be present in propolis extracts. Therefore, the absence or low abundance of a compound in the present dataset should not be interpreted as evidence of its actual absence from the extracts. This limitation may have also constrained the identification of additional chemical compounds, potentially contributing to the observed antioxidant and antibacterial activities. Future studies employing a broader, untargeted or semi-targeted LC-MS/MS metabolomic approach, followed by quantitative confirmation of additional candidate compounds, could provide a more comprehensive characterization of the phytochemical composition and might help to better elucidate the individual and synergistic contributions of phenolic compounds to the biological activities of Turkish propolis.

## Figures and Tables

**Table 1:** LC-MS-based concentrations of selected flavonoids and phenolic acids in the propolis extracts (mg/g) (±%5).

**Phenolic** **Compounds**	**K1**	**K2**	**K3**	**K4**	**K5**	**K6**	**K7**	**K8**	**K9**	**K10**	**K11**	**K12**	**K13**	**K14**	**K15**
**Flavonoids**															
Catechin	3.330	1.203	2.783	1.118	6.802	1.063	0.911	1.602	0.906	1.297	1.348	0.096	3.432	0.608	0.828
Rutin trihidrat	0.106	0.369	0.369	0.112	0.789	0.056	0.166	0.086	0.173	0.024	0.034	0.195	0.224	0.066	0.052
Myricetin	0.087	0.020	0.054	0.018	0.033	0.010	0.023	0.009	0.024	0.005	0.011	0.029	0.231	0.025	0.012
Naringenin	0.478	0.451	0.539	0.436	0.358	0.340	0.336	0.295	0.495	0.030	0.137	0.287	0.070	0.587	0.126
Quercetin	3.283	2.385	5.052	3.377	1.392	3.998	3.349	2.336	3.637	0.110	1.172	2.818	8.519	4.489	0.533
Luteolin	0.403	0.222	0.697	0.897	0.328	0.103	0.670	0.103	0.415	-----	0.085	0.187	1.419	0.404	0.004
Chrysin	0.073	0.066	0.071	0.070	0.087	0.074	0.093	0.060	0.093	0.026	0.066	0.043	0.029	0.035	0.051
Apigenin	0.101	0.071	0.184	0.234	0.136	0.050	0.106	0.037	0.171	-----	0.044	0.050	0.266	0.120	0.008
**Phenolic acids and derivatives**															
Cinnamic acid	5.912	9.562	3.070	34,051	20,608	5.213	10,797	3.188	2.300	1.218	2.436	34,713	12,463	8.881	2.404
Gallic acid	0.017	0.018	0.072	0.085	0.027	0.114	0.018	0.050	0.055	0.024	1.338	0.019	0.044	0.053	0.008
Caffeic acid	25,184	43,345	57,542	172,966	71,025	15,693	36,087	35,952	64,072	8.312	7.569	91,633	9.903	81,535	18,728
2-5 dihydroxy benzoic acid	0.016	0.010	0.022	0.002	0.008	0.012	0.010	0.009	0.011	0.020	0.028	0.006	0.060	0.006	0.010
Trans ferulic acid	4.026	19,666	14,107	15,526	5.569	16,222	17,324	21,629	14,490	7.106	17,078	11,307	2.724	4.150	20,144
Ellagic acid	1.507	2.226	4.784	2.881	1.306	1.087	1.390	1.610	1.896	0.021	2.640	0.983	3.007	3.919	0.513
CAPE	1.369	0.816	0.780	0.404	0.855	0.905	1.205	0.465	0.943	0.150	0.347	0.809	0.208	0.837	0.490
Triacetin	0.011	-----	-----	-----	0.004	-----	-----	-----	-----	-----	0.016	0.004	-----	0.003	-----
**Phenolic Compounds**	**K16**	**K17**	**K18**	**K19**	**K20**	**K21**	**K22**	**K23**	**K24**	**K25**	**K26**	**K27**	**K28**	**K29**	**K30**
**Flavonoids**															
Catechin	0.489	0.738	0.416	3.268	1.912	0.044	0.978	1.023	0.662	6.287	0.096	0.321	0.421	5.878	0.576
Rutin trihidrat	0.190	0.145	0.084	0.322	1.029	0.124	0.081	0.160	0.026	1.007	0.244	0.218	0.136	0.141	0.419
Myricetin	0.010	0.094	0.012	0.012	0.120	0.025	0.018	0.022	0.235	0.045	0.019	0.020	0.015	0.010	0.025
Naringenin	0.396	0.178	0.585	0.656	0.207	0.428	0.292	0.469	0.241	0.378	0.574	0.382	0.378	0.315	0.493
Quercetin	4.534	4.866	4.330	4.737	4.505	3.620	2.199	4.724	3.550	9.145	2.460	2.321	2.869	1.853	5.111
Luteolin	0.149	0.350	0.337	0.238	0.314	0.408	0.080	0.379	0.640	0.629	0.530	0.190	0.219	0.076	0.535
Chrysin	0.059	0.079	0.098	0.073	0.055	0.070	0.061	0.071	0.053	0.072	0.136	0.056	0.054	0.078	0.086
Apigenin	0.071	0.046	0.191	0.153	0.057	0.091	0.033	0.187	0.091	0.210	0.341	0.069	0.091	0.045	0.292
**Phenolic acids and derivatives**															
Cinnamic acid	50.364	5.425	10.857	9.588	1.055	25.396	7.665	1.887	9.051	6.080	4.481	10.033	11.608	4.650	9.352
Gallic acid	0.154	0.037	0.006	0.038	0.028	0.013	0.033	0.019	0.024	0.032	0.030	0.018	0.066	0.099	0.039
Caffeic acid	46.920	30.609	39.584	62.730	35.828	50.470	50.717	57.137	30.805	49.123	15.363	56.978	67.548	23.843	26.645
2-5 dihydroxy benzoic acid	0.014	0.018	0.004	0.006	0.035	0.008	0.013	0.010	0.035	0.018	0.018	0.012	0.006	0.010	0.011
Trans ferulic acid	19.252	17.640	6.914	10.008	12.407	25.571	34.150	5.230	13.879	8.699	1.484	17.307	6.051	19.978	5.701
Ellagic acid	0.541	1.469	4.442	1.677	1.651	1.424	0.297	2.302	1.395	2.739	2.320	0.492	2.742	2.677	4.804
CAPE	0.680	0.649	1.184	1.379	0.564	0.637	0.590	0.658	0.466	0.639	0.587	0.762	0.638	0.846	1.213
Triacetin	0.005	-----	-----	0.003	0.003	-----	-----	-----	-----	-----	-----	-----	-----	-----	-----

-----: Not detected.

**Table 2:** Antibacterial profile of the extracts ZI (mm) MIC and MBC in mg/mL.

	***S. aureus* (+)**			***E. casseliflavus* (+)**			***E. faecalis* (+)**			***P. fluorescens* (−)**		
**Extracts**	**ZI**	**MIC**	**MBC**	**ZI**	**MIC**	**MBC**	**ZI**	**MIC**	**MBC**	**ZI**	**MIC**	**MBC**
**K1**	16.5	0.31	2.5	9.5	0.31	**2.5**	9	0.08	**2.5**	12.5	0.04	**NA**
**K2**	15	0.31	2.5	9.5	0.31	**2.5**	9	0.04	**2.5**	12	0.31	**NA**
**K3**	17	0.63	2.5	10	0.31	**2.5**	8.5	0.63	**2.5**	12	0.31	**NA**
**K4**	13.5	0.31	**2.5**	9	0.31	**2.5**	8	**NA**	**NA**	10.5	0.63	**NA**
**K5**	17	0.31	2.5	9	0.31	**2.5**	8	0.04	**2.5**	11	0.04	**NA**
**K6**	14	0.08	2.5	8	0.31	**2.5**	7	0.04	**2.5**	11	0.16	**NA**
**K7**	15	0.31	2.5	10	0.31	**2.5**	10	0.02	**2.5**	12	0.63	**NA**
**K8**	15.5	0.31	**NA**	8.5	0.31	**2.5**	8.5	0.31	**2.5**	12.5	0.31	**NA**
**K9**	16.5	0.31	**NA**	9	0.31	**2.5**	9	0.16	**2.5**	11	0.31	**NA**
**K10**	10.5	0.63	**2.5**	**NA**	0.31	**2.5**	**NA**	**NA**	**NA**	9.5	0.63	**NA**
**K11**	12	0.63	**2.5**	8.5	0.31	**2.5**	**NA**	**NA**	**NA**	9	0.63	**NA**
**K12**	16.5	0.31	**2.5**	11	0.16	**2.5**	10	0.02	**NA**	12.5	0.08	**NA**
**K13**	18	0.31	**2.5**	10.5	0.31	**2.5**	10	0.08	**2.5**	12.5	0.31	**NA**
**K14**	16	0.31	**2.5**	8	0.31	**2.5**	8	0.16	**2.5**	11	0.08	**NA**
**K15**	15.5	0.31	**2.5**	9.5	0.31	**2.5**	12	0.08	**2.5**	14	0.08	**NA**
**K16**	16.5	0.31	**2.5**	8	0.16	**2.5**	8	0.63	**2.5**	11	0.02	**NA**
**K17**	16.5	0.31	**2.5**	11	0.16	**2.5**	10	0.02	**2.5**	13.5	0.08	**NA**
**K18**	11.5	0.63	**2.5**	7	0.63	**2.5**	**NA**	0.08	**2.5**	9	0.63	**NA**
**K19**	12.5	0.31	**2.5**	10.5	0.31	**2.5**	9.5	0.08	**2.5**	10.5	0.16	**NA**
**K20**	13	0.63	**2.5**	8	0.63	**2.5**	9	0.31	**2.5**	10	0.31	**NA**
**K21**	17.5	0.31	**2.5**	10	0.63	**2.5**	10.5	0.63	**2.5**	14	0.08	**NA**
**K22**	18.5	0.31	**NA**	12	0.63	**2.5**	10	0.63	**2.5**	13.5	0.16	**NA**
**K23**	14	0.31	**2.5**	9.5	0.31	**2.5**	10	0.02	**2.5**	11	0.08	**NA**
**K24**	14	0.63	**NA**	9	0.31	**2.5**	9	0.02	**2.5**	12	0.31	**NA**
**K25**	14	0.31	**2.5**	10.5	0.16	**2.5**	9	0.02	**2.5**	15	0.16	**2.5**
**K26**	14	0.63	**2.5**	9	0.31	**2.5**	9	0.08	**2.5**	10.5	0.63	**NA**
**K27**	15.5	0.31	**2.5**	9	0.16	**2.5**	10	0.08	**2.5**	12	0.31	**NA**
**K28**	15	0.31	**2.5**	10	0.16	**2.5**	10	0.16	**2.5**	12.5	0.31	**NA**
**K29**	15	0.31	**2.5**	10	0.16	**2.5**	7	0.08	**2.5**	10	0.04	**NA**
**K30**	6.5	0.63	**NA**	9	0.63	**2.5**	9.5	0.08	**NA**	8.5	0.63	**NA**
***P. aeruginosa* (−)**						***E. hormaechei* (−)**						
	**ZI**		**MIC**		**MBC**			**ZI**		**MIC**		**MBC**
**K16**	9.5		0.625		2.5	**K15**		10		0.3125		2.5
**K25**	9		0.15625		2.5	**K30**		9.5		0.625		2.5
**K27**	6.5		0.15625		2.5							
**K28**	9		0.625		2.5							
**K30**	11		0.625		2.5							
***S. kentucky* (−)**						***E. aerogenes* (−)**						
	**ZI**		**MIC**		**MBC**			**ZI**		**MIC**		**MBC**
**K12**	8.5		0.15625		2.5	**K9**		9		0.3125		2.5
**K13**	9		0.3125		2.5	**K10**		7		0.625		2.5
**K16**	7		0.3125		2.5	**K12**		9		0.078125		2.5
**K27**	8		0.625		2.5	**K14**		8		0.15625		2.5
**K28**	8		0.3125		2.5	**K15**		8		0.625		2.5
						**K16**		8		0.3125		2.5
***S. typhimurium* (−)**						***S. infantis* (−)**						
	**ZI**		**MIC**		**MBC**			**ZI**		**MIC**		**MBC**
**K12**	8		0.15625		2.5	**K16**		8		0.625		2.5
**K27**	7		0.3125		2.5	**K21**		8.5		0.3125		2.5
**K28**	7		0.3125		2.5	**K30**		8.5		0.625		2.5

ZI: Zone of inhibition; MIC: Minimum inhibitory concentration; MBC: Minimum bactericidal concentration.

**Table 3:** Total phenolic and flavonoid contents, and FRAP and DPPH capacities of the propolis extracts (±%5).

Code	TFC QE mg/g	TPC mg GA/g	FRAP mg AA/g	DPPH %
K1	192.81	76.35	42.95	75.59
K2	321.58	89.41	58.03	84.70
K3	310.85	91.69	65.16	84.50
K4	493.28	27.09	10.23	68.92
K5	486.13	92.92	71.61	84.50
K6	332.31	73.55	42.06	86.10
K7	339.47	62.59	54.95	83.70
K8	232.15	102.04	54.26	85.50
K9	421.74	95.11	56.34	84.70
K10	325.16	7.71	7.56	82.40
K11	604.17	60.66	39.88	70.91
K12	1030.39	191.27	119.40	79.41
K13	839.69	144.11	98.88	80.24
K14	153.46	99.67	64.97	81.91
K15	221.42	46.02	36.01	87.80
K16	436.05	51.98	34.53	57.63
K17	350.20	57.33	45.73	81.80
K18	303.70	48.74	30.36	85.00
K19	260.77	98.88	51.38	85.80
K20	232.15	113.17	60.70	86.30
K21	135.57	52.42	30.36	79.73
K22	1101.91	127.63	88.86	85.20
K23	589.87	143.32	88.86	80.51
K24	1095.95	165.59	107.40	76.85
K25	185.65	93.80	52.47	83.58
K26	275.08	24.63	15.19	53.25
K27	364.51	32.26	23.92	79.46
K28	774.14	126.67	95.31	79.87
K29	368.09	76.88	50.89	81.80
K30	235.73	12.36	12.12	11.32

## Data Availability

The original contributions presented in this study are included in the article/Supplementary Material. Further inquiries can be directed to the corresponding author.

## Supplementary Materials

The following supporting information can be downloaded at: https://www.mdpi.com/article/10.3390/molecules31173071/s1, Table S1: Palynology composition of the propolis samples; Table S2: PCA loadings.
