# Supplementary material for: Comparative Phytochemical Characterization of Turkish Propolis Extracts and Their Antioxidant, Antibacterial, and Anticancer Activities
Source: Molecules. 2026 Aug 31;31(17):3071. doi: 10.3390/molecules31173071 (PMC13567232; doi:10.3390/molecules31173071)
Supplement: Supplementary file 1 [file molecules-31-03071-s001.zip › molecules-4444391-supplementary.pdf]

Comparative Phytochemical Characterization of Turkish Propolis Extracts and Their Antioxidant, Antibacterial, and Anticancer Activities

Serhat Karabıcak[1], Oktay Bıyıklıoğlu[1*], Şeymanur Aktaş[1], Derya Keleşoğlu Kalemkaş[1], Demet Erdağ[2], Tarık Mecit[3], Selin Çeter[1], İdris Yazgan[1], Talip Çeter[1]

[1]Department of Biology, Faculty of Science, Kastamonu University, Kastamonu 37150, Türkiye

[2]Department of Biophysics, Faculty of Medicine, Bilecik Seyh Edebali University, Bilecik 11100, Türkiye

[3]Department of Physiology, Faculty of Medicine, Biruni University, İstanbul 34025, Türkiye

*Corresponding Author: oktayman@gmail.com

Table S1: Palynology composition of the propolis samples.

Plant Family	K1	K2	K3	K4	K5	K6	K7	K8	K9
**Amaranthaceae**	T	-	-	-	T	-	T	-	-
**Amaryllidaceae**	T	-	-	-	-	-	-	-	-
**Apiaceae**	M	M	M	T	M	M	M	T	M
**Araliaceae**	M	M	T	T	T	M	M	T	M
**Asparagaceae**	T	-	-	T	-	-	T	T	T
**Asteraceae**	M	M	T	T	M	M	M	T	M
**Boraginaceae**	M	M	T	T	M	M	M	T	M
**Brassicaceae**	T	-	T	T	T	T	M	-	T
**Caryophyllaceae**	-	T	-	-	-	T	T	-	-
**Cisteraceae**	-	-	T	-	T	-	T	-	T
**Cornaceae**	T	T	T	T	T	T	T	T	T
**Campanulaceae**	T	T	-	T	-	T	T	-	T
**Tricaceae**	-	-	-	-	T	T	T	T	-
**Fabaceae**	M	D	M	D	D	D	D	M	D

**Fagaceae**	D	S	S	M	M	M	M	D	S
**Lamiaceae**	M	T	T	T	T	T	T	M	T
**Myrtaceae**	T	-	-	-	-	-	-	T	-
**Malvaceae**	M	T	T	T	T	M	M	T	T
**Moraceae**	T	T	T	T	T	T	T	M	M
**Papaveraceae**	-	-	T	T	-	T	T	M	T
**Polygalaceae**	T	-	T	T	T	-	T	-	T
**Pinaceae**	T	T	T	T	T	T	T	T	-
**Poaceae**	T	-	T	-	T	T	T	-	-
**Ranunculaceae**	-	T	-	T	T	T	T	-	T
**Rosaceae**	T	M	D	T	T	T	T	S	M
**Salicaceae**	T	-	T	T	-	M	-	T	-
**Ulmaceae**	T	T	T	T	T	T	-	T	-

Table S1 Continuing

**Plant Family**	**K10**	**K11**	**K12**	**K13**	**K14**	**K15**	**K16**	**K17**	**K18**
**Amaranthaceae**	-	-	T	-	-	-	-	-	-
**Amaryllidaceae**	-	-	-	T	-	-	-	-	T
**Apiaceae**	T	M	T	T	M	M	M	M	M
**Araliaceae**	T	M	M	M	M	M	M	M	M
**Asparagaceae**	T	T	-	-	-	-	T	T	T
**Asteraceae**	T	-	T	M	M	M	M	M	T
**Boraginaceae**	T	M	M	M	M	M	M	M	M
**Brassicaceae**	T	T	M	-	M	T	T	-	T
**Caryophyllaceae**	T	-	T	T	-	T	-	T	T
**Cisteraceae**	-	T	T	-	T	-	-	T	-

**Cornaceae**	T	T	T	T	T	T	T	T	T
**Campanulaceae**	T	-	T	-	-	-	T	T	-
**Tricaceae**	-	T	-	-	M	T	T	-	-
**Fabaceae**	D	S	S	S	M	S	S	D	D
**Fagaceae**	M	D	M	M	T	D	M	M	M
**Lamiaceae**	T	M	T	M	T	T	T	T	M
**Myrtaceae**	-	-	-	-	T	T	-	-	-
**Malvaceae**	T	M	T	M	T	M	T	M	M
**Moraceae**	T	T	T	T	M	M	-	-	-
**Papaveraceae**	T	-	T	T	-	-	-	-	-
**Polygalaceae**	-	T	T	T	-	-	T	-	T
**Pinaceae**	T	-	T	T	T	M	M	M	M
**Poaceae**	T	-	T	T	-	-	-	-	T
**Ranunculaceae**	-	-	T	T	M	-	T	T	T
**Rosaceae**	T	M	D	D	D	M	D	T	T
**Salicaceae**	T	-	-	T	T	-	-	T	-
**Ulmaceae**	T	-	-	T	T	-	T	-	-

Table S1 Continuing

**Plant Family**	**K19**	**K20**	**K21**	**K22**	**K23**	**K24**	**K25**	**K26**	**K27**	**K28**	**K29**	**K30**
**Amaranthaceae**	-	-	-	T	-	T	T	T	T	T	M	M
**Amaryllidaceae**	-	T	T	-	T	-	-		-	T	T	T
**Apiaceae**	T	M	T	T	M	M	T	M	M	M	S	M
**Araliaceae**	T	M	T	M	M	M	M	M	M	M	S	M
**Asparagaceae**	-	-	-	-	T	T	-	-	-	T	T	T
**Asteraceae**	T	M	T	M	M	T	M	M	M	M	S	M
**Boraginaceae**	-	M	M	M	M	M	M	M	M	M	S	M
**Brassicaceae**	T	T	T	T	M	T	M	M	M	M	S	M

**Caryophyllaceae**	-	T	-	T	-	T	T	T	T	T	T	M
**Cisteraceae**	-	T	-	T	-	T	-	-	T	T	-	-
**Cornaceae**	T	T	T	T	T	T	-	T	T	-	T	T
**Campanulaceae**	-	T	-	T	T	T	-	T	T	-	-	-
**Tricaceae**	-	-	-	-	-	-	T	T	T	-	-	-
**Fabaceae**	D	S	S	D	D	D	M	M	S	S	D	D
**Fagaceae**	M	D	M	M	M	M	D	D	D	D	S	M
**Lamiaceae**	M	T	T	M	M	T	T	T	M	M	S	M
**Myrtaceae**	-	T	-	T	-	-	-	T	-	-	-	-
**Malvaceae**	T	M	T	M	M	M	M	M	M	M	M	M
**Moraceae**	M	T	T	T	T	T	T	T	T	-	T	
**Papaveraceae**	T	T	-	T	T	-	M	T	T	T	M	T
**Polygalaceae**	-	T	T	T	T	T	T	T	T	-	T	-
**Pinaceae**	T	T	T	M	M	M	T	M	T	M	T	T
**Poaceae**	T	-	T	T	T	T	T	T	-	-	-	T
**Ranunculaceae**	T	T	T	T	T	T	M	T	M	M	M	M
**Rosaceae**	S	M	D	S	T	S	S	T	M	M	S	S
**Salicaceae**	T	T	-	T	T	-	T	T	T	T	M	M
**Ulmaceae**	T	T	T	-	-	T	T	T	T	T	-	T

Table S2. PCA loadings

Parameters	PC 1	PC 2
TFC QE mg/g	**0.31457**	0.1078
TPC mg GA/g	0.28239	0.21802
FRAP mg AA/g	0.29664	0.21376
DPPH %	0.23093	-0.059835
Catechin	-0.0048153	0.1609
Cinnamic acid	-0.02379	-0.01407
Gallic acid	0.093177	-0.11354
Caffeic acid	-0.10737	0.028664
2-5 dihydroxy benzoic acid	0.2579	0.29331
Trans ferulic acid	0.15036	-0.27099
Rutin trihidrat	-0.082381	0.20912
Myrcetin	0.23594	**0.35534**

Naringenin	-0.38225	0.049418
Ellagic acid	-0.2486	0.25385
Quercetin	-0.076131	**0.39526**
Luteolin	-0.05164	**0.42379**
Chrysin	-0.33612	-0.0086893
Apigenin	-0.29669	**0.33478**
CAPE	-0.29322	-0.011837
Triacetin	0.065207	-0.085442
